# Achromatic vector vortex beams from a glass cone

**DOI:** 10.1038/ncomms10564

**Published:** 2016-02-10

**Authors:** N. Radwell, R. D. Hawley, J. B. Götte, S. Franke-Arnold

**Affiliations:** 1 SUPA and School of Physics and Astronomy, University of Glasgow, Kelvin Building, Renfrewshire, Glasgow G12 8QQ, UK; 2 Max Planck Institute for the Physics of Complex Systems, Nöthnitzer Strasse 38, 01187 Dresden, Germany

## Abstract

The reflection of light is governed by the laws first described by Augustin-Jean Fresnel: on internal reflection, light acquires a phase shift, which depends on its polarization direction with respect to the plane of incidence. For a conical reflector, the cylindrical symmetry is echoed in an angular variation of this phase shift, allowing us to create light modes with phase and polarization singularities. Here we observe the phase and polarization profiles of light that is back reflected from a solid glass cone and, in the case of circular input light, discover that not only does the beam contain orbital angular momentum but can trivially be converted to a radially polarized beam. Importantly, the Fresnel coefficients are reasonably stable across the visible spectrum, which we demonstrate by measuring white light polarization profiles. This discovery provides a highly cost-effective technique for the generation of broadband orbital angular momentum and radially polarized beams.

## Abstract (web summary)

Under total internal reflection light acquires a phase shift that depends on its polarisation. Here, the authors show that this effect can be harnessed to generate so-called vector vortex beams—light with polarization and phase singularities—when white light is back-reflected from a glass cone

## Graphical abstract

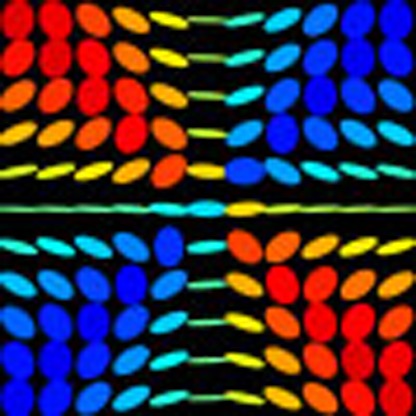

Optics is possibly the oldest scientific discipline and basic ideas of reflection and refraction were already mentioned in a treatise by Ptolemy in the second century AD^1^. Light has been harnessed for hundreds of years for applications ranging from microscopy to astronomy. The full vectorial nature of electromagnetic radiation, however, has a far shorter history and its use for imaging and sensing is still largely unexplored. Of particular interest are light beams with phase and polarization singularities; the former, associated with twisted phasefronts, carry orbital angular momentum (OAM) and the latter are known as vector vortex beams. They are associated with an azimuthally varying phase shift between their orthogonal polarization components or with a correlation between OAM and spin angular momentum^2^.

OAM is defined by the rate at which phasefronts spiral around the beam axis: an azimuthal phase exp(*imφ*) results in a wavefront tilt of 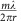 and an OAM of *mħ* per photon. This implicit wavelength dependence makes generation of white light vector vortex beams technically challenging^3^, as most currently available techniques rely on refractive elements: forked holograms, astigmatic mode converters and *q*-plates^4^.

Vector vortex beams have structured polarization and have received attention for their ability to contain fields unobtainable with uniform polarization^5^. The spiralling polarization structures of these beams^6^,^7^ are associated with OAM and have been used to demonstrate Moebius band-like topologies^8^,^9^,^10^. Such beams are structurally inseparable in polarization and angular position^11^,^12^,^13^,^14^,^15^,^16^. Radially polarized laser beams contain strong longitudinal fields in the focal region^17^,^18^,^19^, creating spot sizes below the conventional diffraction limit^20^, which are of interest for applications from optical lithography and material processing to super-resolution imaging. Current generation methods include commercial liquid crystal devices, segmented waveplates^21^, *q*-plates^22^, interferometric methods^23^ and conical intra-cavity prisms^24^.

In this study, we demonstrate the generation of broadband phase and polarization singularities, including radial polarization. Our technique is based on applying a geometric Pancharatnam–Berry phase^25^, which is intrinsically wavelength independent. Experimentally, we realize this geometric phase by back-reflecting light from a solid glass cone with a 90° apex angle (for illustration, see Fig. 1a. The idea that conical back reflection can lead to the generation of OAM was recently investigated theoretically^26^ and demonstrated for the case of laser light reflected from a concave conical mirror^27^. Here we generate and measure white-light OAM from total internal reflection (TIR) within a solid glass cone. Moreover, we show that the Fresnel coefficients associated with TIR at the glass–air interface result in polarization singularities. In turn, these can be easily converted to produce broadband radially or azimuthally polarized beams. This presents a highly cost-effective method with benefits for short pulse and other broadband applications.

## Results

### Jones matrix calculus for conical back reflection

In the following we show that two effects are relevant for back reflection off a glass cone: the geometric phase or spin redirection phase and a Fresnel-induced phase. The effect of the geometric phase results in a conversion of spin to OAM^27^, an argument that holds for hollow metallic and solid glass cones as well: this can be understood by considering the conservation of angular momentum during the backscattering process. Reflection converts right circularly polarized light into left circularly polarized light and vice versa. The conical mirror imposes two reflections, so that circularly polarized light leaves in the same polarization state. However, as the propagation direction of the light is reversed, this corresponds to a change in angular momentum of ±2*ħ*. Owing to the cylindrical symmetry, no angular momentum can be transferred from light to cone and the change of ±2*ħ* is compensated by a change of OAM of ±2*ħ*. These arguments are based on considerations in ref. 26, have been discussed in ref. 27 for metallic reflection and are also supported by the theory presented in this study. We note that similar devices can also be realized in transmission^28^.

**Figure 1:**
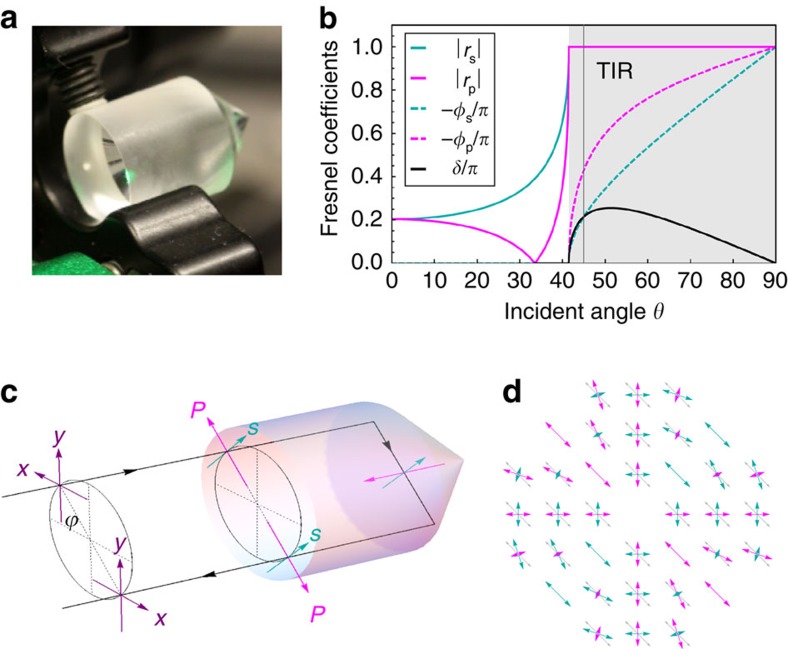
Total internal reflection from a solid glass cone. (**a**) Photo of a solid glass cone. (**b**) Fresnel coefficients and phase shift (black line), assuming *n*=1.51. (**c**) Illustration of the axis conventions in the (*x*,*y*) and (p,s) bases. (**d**) Owing to the rotational cone symmetry, the decomposition of a linear polarized input beam (grey) into its s and p components (cyan and magenta, respectively) varies across the beam.

To predict the polarization structure arising from reflection by the glass cone, we follow the Jones calculus approach presented in ref. 29. In geometric optics, each ray will experience two internal reflections before exiting the cone again. The Fresnel reflection coefficients for the vector components perpendicular (s) and parallel (p) to the plane of incidence are


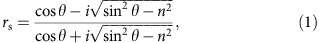



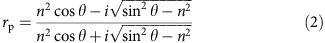


where *n*=*n*_air_/*n*_glass_<1 is the ratio of refractive indices and *θ* is the angle of incidence. For a cone with a 90° apex angle and a light beam in the direction of its symmetry axis, the incidence angle for both internal reflections is *θ*=*π*/4. The vertical grey line in Fig. 1b illustrates that for our glass cone, *θ* is above the critical angle for TIR, so that the reflections affect only the optical phase. At each interface, the s and p polarization components acquire a differential phase shift of


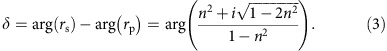


Two subsequent reflections are then described by the Jones matrix


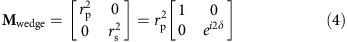


expressed in the (p,s) eigensystem, as illustrated in Fig. 1c. The prefactor of 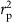 constitutes an overall phase, which does not influence intensity and Stokes measurements.

For a standard glass cone (N-BK7 @ 780 nm, *n*_glass_=1.512), the total phase shift is 2*δ*=0.44*π*. This is close to *π*/2 (which would require *n*_glass_=1.5538), so that the cone works effectively similar to a quarter waveplate for the s and p polarization components. For a linearly polarized input beam, say at 45°, the polarization direction is purely p-polarized at azimuthally opposed angles along the diagonal axis and purely s-polarized along the antidiagonal axis, illustrated in Fig. 1d. At these four angles, the polarization therefore remains linear after back reflection. However, along the horizontal and vertical angles, the field has equal components of p and s polarization, and the cone acts similar to a quarter waveplate, resulting in a conversion to (nearly) left and right circular polarization.

Mathematically, the Jones matrix of the cone can be constructed by rotating the input light field (*E*_*x*_, *E*_*y*_) expressed in the (*x*, *y*) basis system into the local (p,s) eigensystem of the cone via the usual rotation matrix 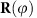, then applying the two reflections and finally rotating back into the (*x*, *y*) basis:


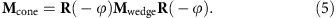


It is note worthy that here we have adopted a right-handed coordinate system, defined with respect to beam propagation. As the back reflection changes the sense of rotation (see Fig. 1c), we need to rotate twice in the same direction.

### Polarization and phase conversion

We find that the Jones matrix simplifies considerably if expressed in terms of a (*σ*_−_, *σ*_+_) circular polarization basis, which is better adapted for the cylindrical symmetry of the cone and for OAM modes as well. The Jones matrix in circular polarization notation reads:


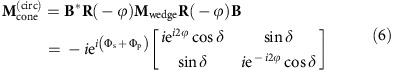


where the unitary matrix **B**=2^−1/2^((1, 1), (−*i*, *i*)) rotates an electric field from the (*σ*_−_, *σ*_+_) basis to the (*x*,*y*) basis and B* is the conjugate transpose. One can immediately identify the hallmark of OAM in the 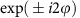 terms. Right-hand circularly polarized light, for example, is converted by the cone such that


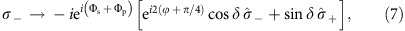


acquiring 2*ħ* units of OAM in the right but none in the left circular component. The total angular momentum, including the spin, is therefore *ħ* for both polarization components, defined in beam direction. It is worth noting that this is in agreement with angular momentum conversion: the angular momentum of the incoming right-handed polarized light was −*ħ* in the direction of the input beam, that is, +*ħ* in the coordinate system defined by the outgoing beam.

In our experiment, the Fresnel-induced phase factor is close to *δ*=*π*/4, so that for circularly polarized input light the back-reflected light contains equal amounts of right- and left-handed polarized light:


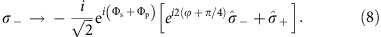


At every position of the beam profile, the polarization is linear but the polarization direction varies with azimuthal angle, resulting in a polarization vortex reminiscent of those observed for *q*-plates of *q*=1/2. Although similar, there is a subtle difference, as in the case of a *q*-plate the phase winding is distributed equally over both circular polarization components, whereas here all of the OAM is contained within a single component leading to a net OAM of 1*ħ* for the beam.

In contrast, for a perfectly conducting metal cone, the phase shift in equation (7) is that of a simple mirror reflection (*δ*=*π*), so that right-hand-polarized input light is converted to right-hand-polarized output light, 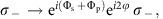 generating an OAM of 2*ħ*, as reported in ref. 27. Interestingly, the OAM of ±2*ħ* present in the right- and left-handed polarization components is associated with the geometric redirection phase, irrespective of the cone's refractive index. The polarisation pattern however depends crucially on the phase shift acquired according to the Fresnel coefficients and thereby on the refractive index of the conic reflector.

**Figure 2:**
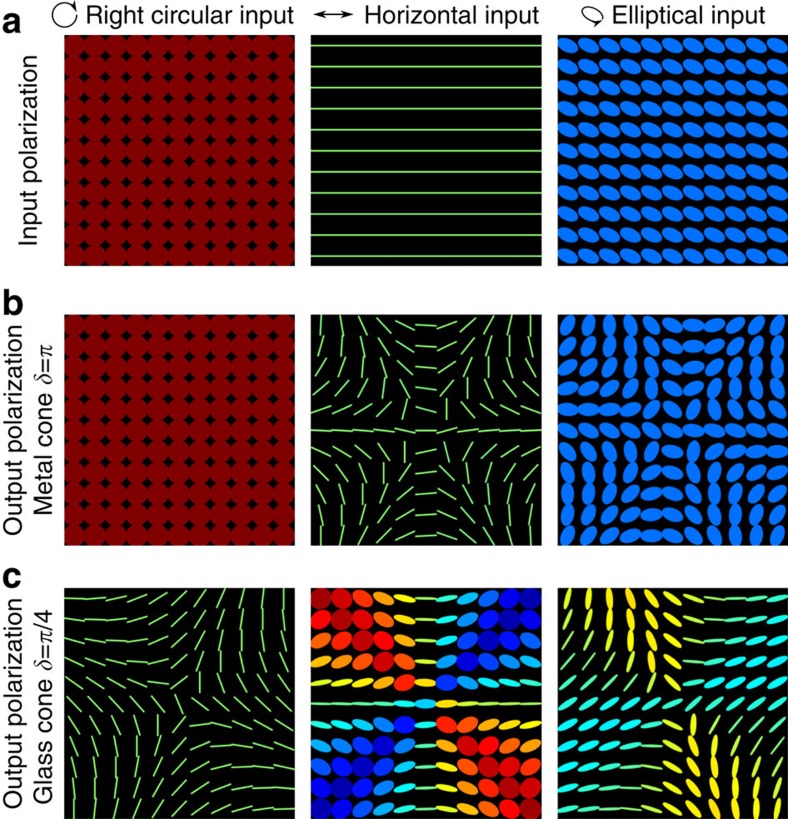
Theoretical polarization patterns. Theoretical prediction of polarization patterns arising from conical back reflection. The polarization ellipse is plotted at 11 × 11 grid positions, with red corresponding to right- and blue to left-hand circular polarisation. (**a**) The input polarization profiles. (**b**) The predicted polarization profile after metallic reflection from a perfectly conducting hollow cone and (**c**) after TIR from an ideal glass cone with a Fresnel-induced phase shift of *π*/4 at each interface.

Different polarization patterns are generated depending on the input polarization, a selection of which are shown in Fig. 2. It is noteworthy that the geometric phase results in an azimuthal variation of the orientation of the output polarization, corresponding to rotations around the equatorial plane of the Poincaré sphere. This is demonstrated by the ideal metallic reflection, shown in Fig. 2b. The differential phase shift induced by the Fresnel equations instead can induce a variation of the ellipticity, giving access to the whole Poincaré sphere. This is the case for TIR experienced in the glass cone, shown in Fig. 2c.

For circular input light, the resulting linear polarization singularity can be converted into a radially or azimuthally polarized light beam simply by transmission through a *λ*/2 plate. To see this, we express the circular polarization components of equation (8) in the (*x*, *y*) basis:


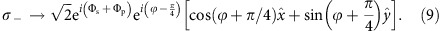


A rotation by −*π*/4 rotates this into radial polarization and by +*π*/4 into azimuthal polarization.

### Experimental realization

**Figure 3:**
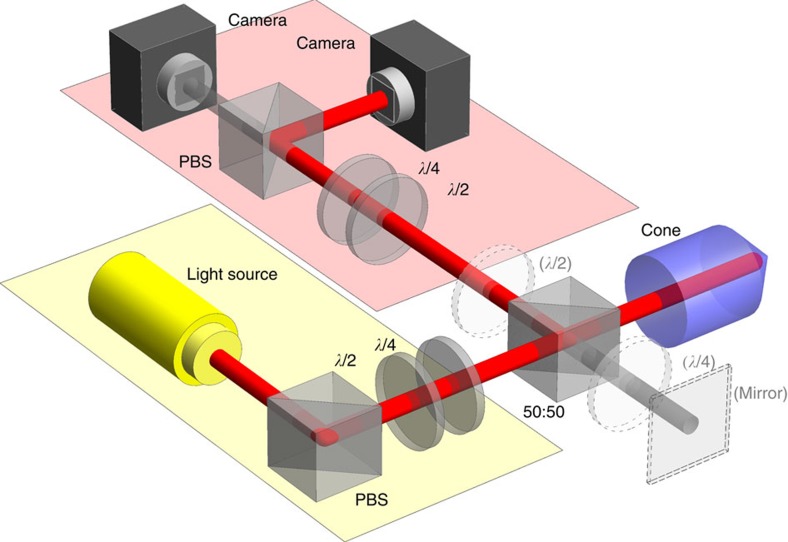
Experimental setup. PBS, polarizing beamsplitter; *λ*/2, half waveplate; *λ*/4, quarter waveplate (for white light, these are Fresnel rhombs). The optional mirror and *λ*/4 may be included to form an interferometer; the optional *λ*/2 plate was used to produce radial and azimuthal polarization.

The experimental setup, shown in Fig. 3, comprises three stages. The first stage prepares the beam in the desired input polarization, which in the second stage is reflected from the cone. The cone we use is an Edmund Optics 45–939 with 10 mm diameter, 15 mm length. The front facet is uncoated and we have removed the metal coating using printed circuit board etching fluid. The final stage measures the intensity of the reflected light in one of the polarization bases, (horizontal and vertical), (antidiagonal and diagonal) or (right and left). Each basis state is measured successively by rotating the output waveplates and recording the intensity profile with a camera, see also Supplementary Figs 1–3 and Supplementary Note 1. This allows us to produce spatially resolved Stokes vectors, revealing the polarization profile across the beam. The lenses that image the cone tip onto the camera have been omitted from Fig. 3 for clarity.

**Figure 4:**
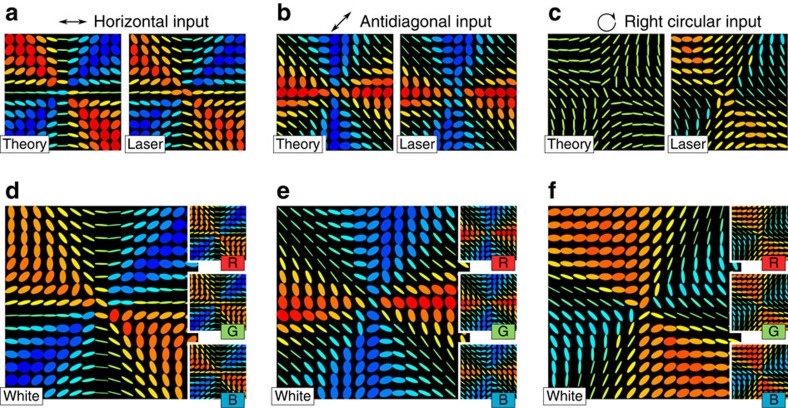
Polarization patterns for light reflected from a solid glass cone polarization patterns. (**a**–**c**) Simulated (left) and experimental (right) polarization patterns produced from reflection of a solid glass cone with the indicated input polarization, using a diode laser at 780 nm. (**d**–**f**) Experimental polarization patterns obtained from a white light LED. The insets show the red, green and blue colour planes of the camera.

Our initial measurements use a spatially filtered 780-nm laser diode as the light source. The right panels of Fig. 4a–c show the output polarization pattern obtained after reflection from the cone when we set the input polarization to be horizontal, antidiagonal and right-hand circular polarized. These are compared with the prediction from a simulation based on equations (5) and (6), modified to allow for the actual refractive index of the NBK-7 glass cone and are shown on the left panel of Fig. 4a–c. Experimentally, the beamsplitter can introduce unwanted phase shifts, which we correct for by measuring its Mueller matrix and multiplying the data by its inverse (for more information, refer Supplementary Fig. 4 and Supplementary Note 2). We find excellent agreement between the experimental data and the simulation, confirming the validity of the model.

As discussed before, these spatially varying polarization structures arise due to phase shifts during TIR, which are intrinsically broadband due to the weakly dispersive nature of glass (*δ*=0.466*π*@400 nm and *δ*=0.435*π*@700 nm). This opens up the possibility to obtain white light polarization and phase structures; we demonstrate this by replacing the laser source in Fig. 3 with a white LED source and waveplates with Fresnel rhombs. The obtained polarization patterns are reported in Fig. 4d–f, which show the polarization structure in the white light and in its individual red, green and blue components as recorded by the camera.

**Figure 5:**
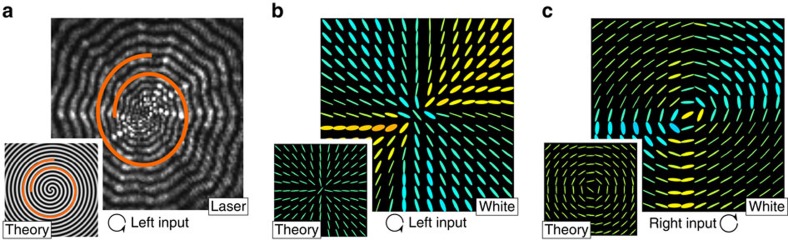
Phase and polarization singularities. (**a**) Interferometric detection of OAM for left-handed input, the inset shows the calculated output for the interference of a beam with 2 units of OAM and a Gaussian. The double spiral indicates the presence of 2*ħ* OAM. (**b**) Radial and (**c**) azimuthal polarization patterns generated by the glass cone followed by a half waveplate. Inset images show the theoretical prediction based on a Jones calculus model.

In the following we will concentrate on the azimuthal phase dependence of the beam profiles generated from circularly polarized input light. This phase dependence is visible in the polarization singularity as well and indicative of the conversion from spin to OAM^4^. Indeed, the observed rotation of the linear polarization direction is due to an azimuthal phase variation between the circular polarization components. To visualize the OAM content we return to the laser light source and employ interferometric techniques: adding the optional mirror and waveplate indicated in Fig. 3 produces a Michelson interferometer, which overlaps the back-reflected cone beam with the circularly polarized input beam. The standard Newton's fringes expected for two Gaussian beams are turned into spiraling fringes, shown in Fig. 5a, and the double spiral structure confirms the OAM value of 2*ħ*. Back reflection of left-hand polarization produces a double spiral structure with the opposite sense of direction (not shown). The fringe spacing is dictated by the small deviation of the cone apex angle from 90° (∼0.35°), which effectively leads to a very slight convergence of the cone beam.

Finally, we demonstrate the ability to produce a broadband radially polarized beam from back reflection off a glass cone. As discussed in association with equation (9), a half wave plate inserted at the output of the system can rotate the linear polarizations shown in Fig. 4f, to create either radial or azimuthal polarization beams. We demonstrate this for a white light beam in Fig. 5b,c, respectively. The deviation of *δ* from the ideal value of *π*/4 for our glass cone results in the slight ellipticity, which is also seen in our simulation data.

## Discussion

We have shown that beautiful light structures arise from an optical system as simple as a glass cone manifest as polarization and phase singularities. We note that our setup has an efficiency of only 25%, owing to passage through a non-polarizing beam splitter. In principle, however, conversion should be 100% and a potential setup is shown in Supplementary Fig. 5 and is discussed in Supplementary Note 3.

We have predicted that spatially varying polarization structures arise in the back-reflected light due to the difference in Fresnel coefficients of local s and p polarization components. Our experimental observations based on spatially resolved Stokes parameter measurements have confirmed these predictions for laser light and even for incoherent white light. For circularly polarized input light, we have shown that TIR generates a superposition of one circular polarization component containing two units of OAM and one without. The associated polarization singularities are topologically equivalent to radial or azimuthal polarized light, allowing us to generate radial polarization of incoherent white light, albeit with different helical phase dependence and hence focusing properties.

This opens up new avenues to produce OAM, position polarization-correlated light and radially polarized beams with far wider bandwidth and orders of magnitude cheaper production costs than alternative techniques. We anticipate applications in white light microscopy including optical manipulation^30^ and sensing^31^,^32^, as well as for optical vortex coronagraphs in astronomy^33^.

## Additional information

**Data deposition:** The data used to generate all of the figures in this study can be found at http://dx.doi.org/10.5525/gla.researchdata.243.

**How to cite this article:** Radwell, N. *et al*. Achromatic vector vortex beams from a glass cone. *Nat. Commun.* 7:10564 doi: 10.1038/ncomms10564 (2016).

## Supplementary Material

Supplementary Information Supplementary Figures 1-5 and Supplementary Notes 1-3.
