# Supplementary material for: Achromatic vector vortex beams from a glass cone
Source: Nat Commun. 2016 Feb 10;7:10564. doi: 10.1038/ncomms10564 (PMC4749967; doi:10.1038/ncomms10564)
Supplement: Supplementary Information — Supplementary Figures 1-5 and Supplementary Notes 1-3. [file ncomms10564-s1.pdf]

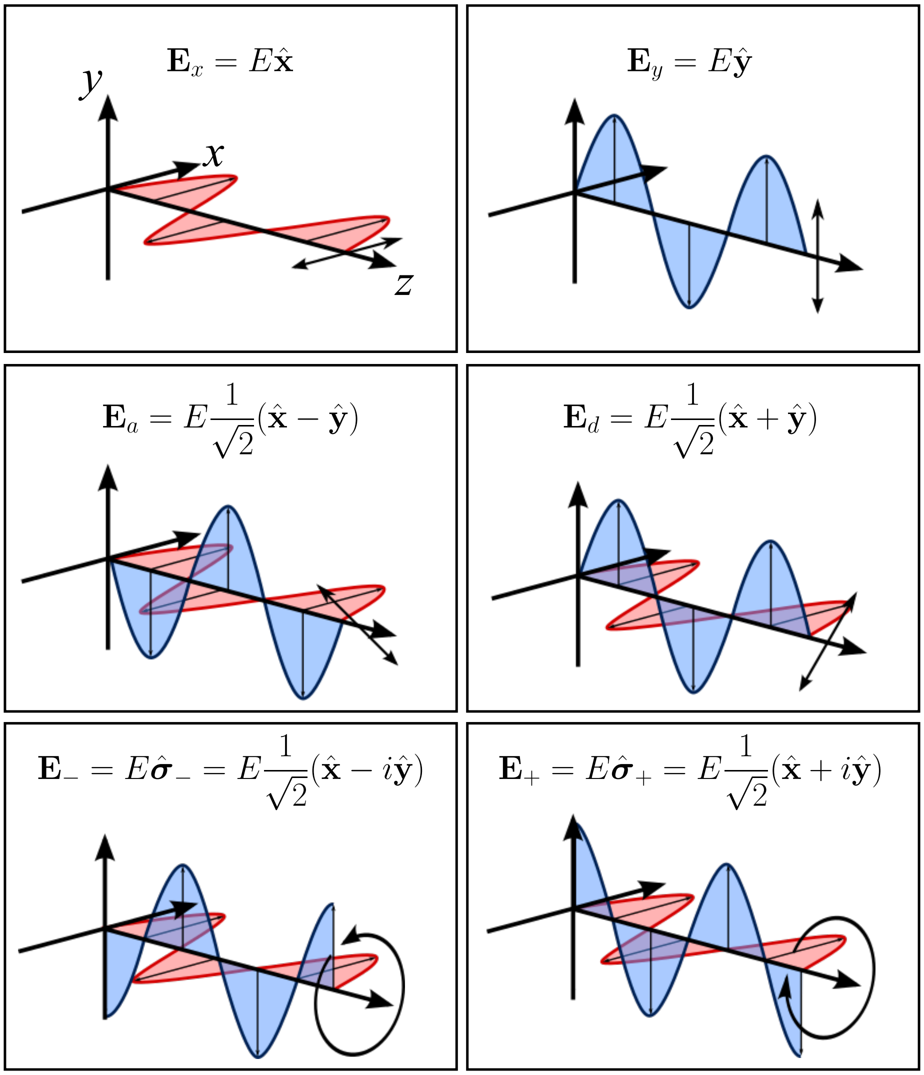

Supplementary Figure 1: **Polarisation basis states** Definition of each of the polarisation basis states.

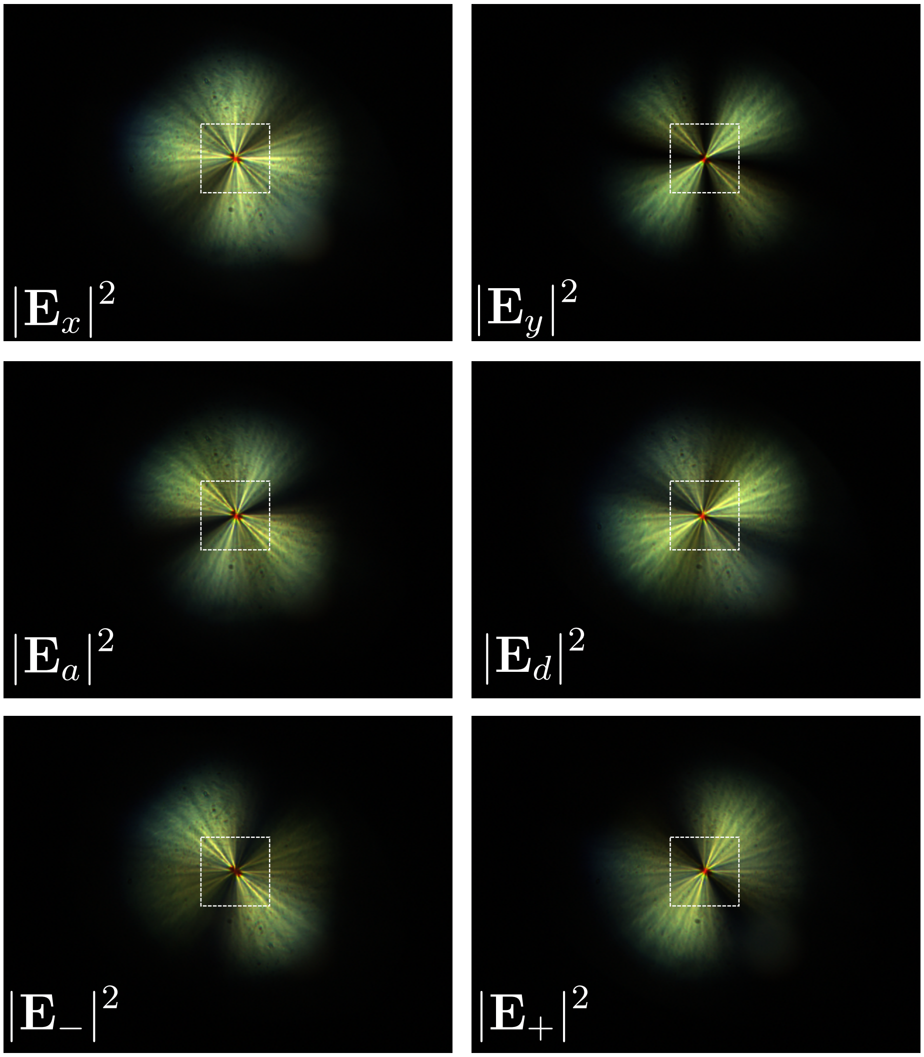

Supplementary Figure 2: **Basis state intensity measurements** Intensities measured in each of the polarisation bases as defined in Supplementary Figure 1. The light source is white light and the input polarisation is set to be horizontal linear polarisation. The dashed line shows the extent of the cropping used in further analysis.

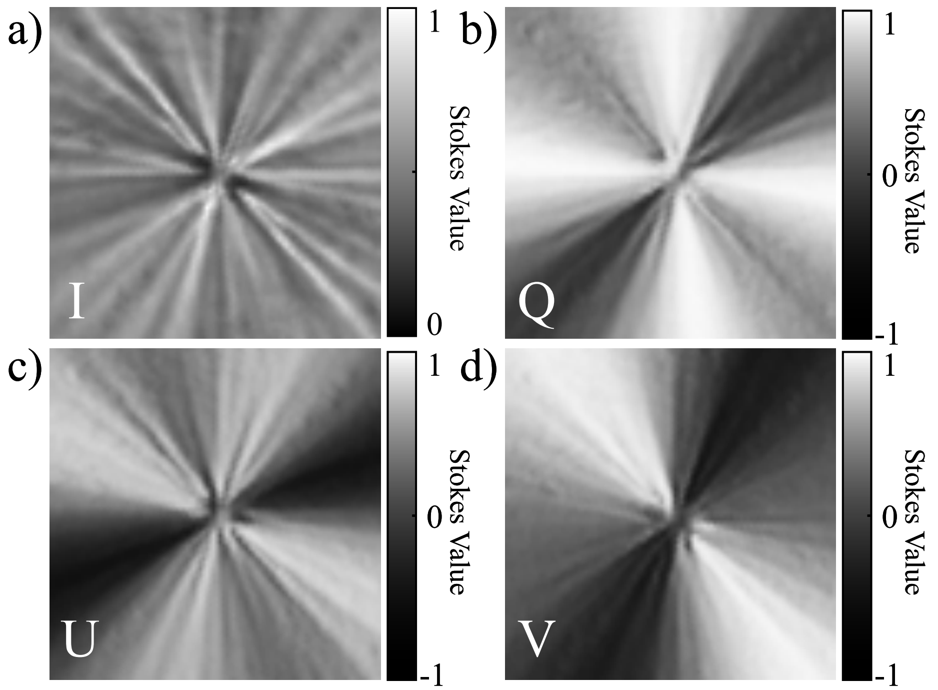

Supplementary Figure 3: **Conversion of intensities to Stokes parameters** (a)-(d) The Stokes vector components for the data shown in Supplementary Figure 2.

(a) (b)

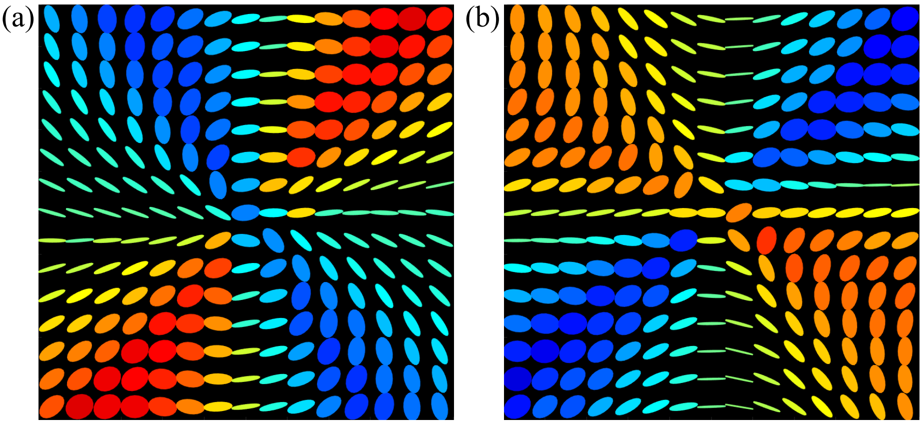

Supplementary Figure 4: **Mueller correction** Example of the polarisation states before and after Mueller correction for the same data as that shown in Supplementary Figure 3.

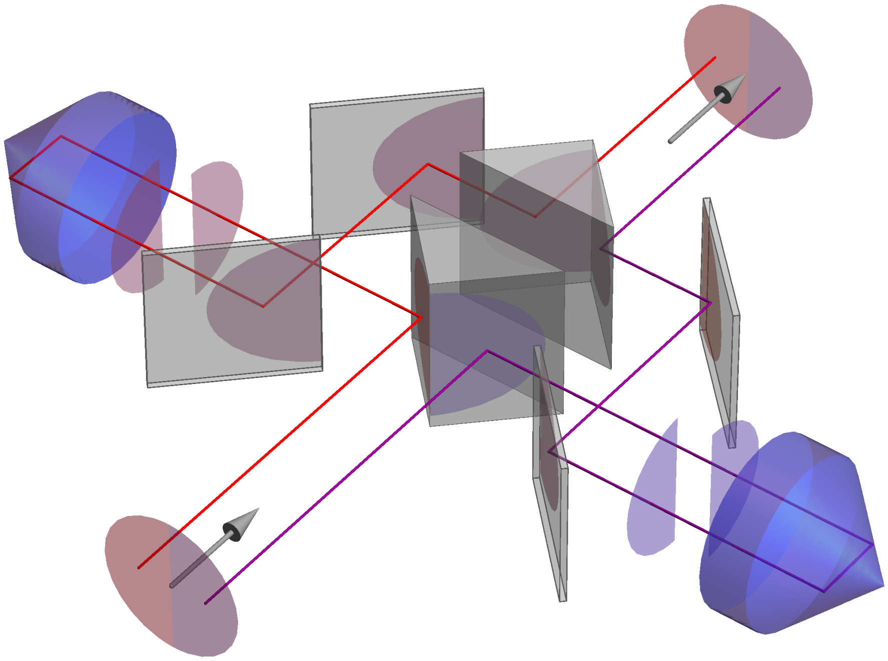

Supplementary Figure 5: **Optimised experimental setup** Potential set-up for optimal efficiency, containing two TIR cones, two (polarisation independent) beam-splitting mirrors and four mirrors.

Supplementary Note 1: Measurement of spatially dependent polarisation states

We measure spatially resolved Stokes vectors by recording the intensity components in six polarisation states. The definition of each of these basis states can be seen in Supplementary Figure 1, note the right-handed basis convention. These measurements are achieved experimentally using a quarter wave retarder and a polariser with the intensity recorded using a camera, identifying $\hat{x}$ with horizontal and $\hat{y}$ with vertical polarisation. The linear polarisation bases are measured simply by rotating a linear polariser. To measure the circular bases, we use an additional quarter wave retarder. An example of the intensities measured in each of these basis states is shown in Supplementary Figure 2 for the case of horizontally polarised input light. These six images are then cropped to a size indicated by the white square centered with the cone axis and converted into spatially resolved Stokes vectors using:

$$I = |E_x|^2 + |E_y|^2 \tag{1}$$
$$Q = |E_x|^2 - |E_y|^2 \tag{2}$$
$$U = |E_a|^2 - |E_d|^2 \tag{3}$$
$$V = |E_-|^2 - |E_+|^2. \tag{4}$$

Here I, Q, U and V are the four elements of the Stokes vector which describe the polarisation state. For illustration, Supplementary Figure 3 shows the components of the spatially resolved Stokes vector based on the measurements of Supplementary Figure 2. To visualise the polarisation state we then convert the Stokes vector into the polarisation ellipse.

The cropped images from the camera have a resolution of 200 × 200 pixels, however for clarity we only want to plot 15 ellipses (chosen as a compromise between resolution and clarity of presentation) and therefore we spatially average the data such that we have a 15 × 15 array of Stokes vectors. For each of the Stokes vectors the conversion to the polarisation ellipse is then performed by introducing the parameters

$$I_p = \sqrt{Q^2 + U^2 + V^2} \tag{5}$$
$$L = Q + iU. \tag{6}$$

The parameters of the polarisations ellipse can then be calculated from

$$A = \sqrt{\frac{1}{2}(I_p + |L|)} \tag{7}$$

$$B = \sqrt{\frac{1}{2}(I_p - |L|)} \tag{8}$$

$$\psi = \frac{1}{2}\arg(L) \tag{9}$$

$$h = \mathrm{sgn}(V), \tag{10}$$

where A and B are the sizes of the major and minor ellipse axes, ψ is the angle through which the major axis of the ellipse is rotated and h indicates the handedness. Supplementary Figure 4(a) shows the conversion to the polarisation ellipses.

Supplementary Note 2: Mueller matrix compensation

The measured intensities in the polarisation states and therefore also the deduced Stokes parameters and polarisation ellipses are not only influenced by the back-reflection from the cone which we want to study, but also by any additional polarisation dependence introduced by other optical elements. In our experiment the main compromising element is a beam splitter cube which imposes a polarisation dependent phase shift on the reflected beam inducing unwanted polarisation rotations on the output beam from the cone, in particular for white light. We note that by using higher quality optical elements these effects may be avoided. Alternatively the effect can be identified and eliminated. In this section we detail the procedure used to do so.

In Jones calculus, polarisation states are expressed as a two-element Jones vector and are acted upon by optical elements described by a 2×2 Jones matrix. Analogously, in a Stokes picture, a polarisation state represented by a four-element Stokes vector is acted on by a 4×4 Mueller matrix. By characterising the Mueller matrix of our beam splitter we can undo its unwanted polarisation rotations and discover the true polarisation states exiting the cone.

The characterisation is performed by placing the optical component between a polariser and analyser. The polariser sets the input polarisation and the analyser sets the polarisation state to measure. Careful choice of the polariser and analyser settings allows us to build up the 16 elements of the Mueller matrix from 18 measurements. This includes 2 extra measurements which are used to normalise the matrix.

Once the Mueller matrix is known it is numerically inverted, such that multiplication with the measured polarisation state results in a corrected result. An example of this correction is shown in Supplementary Figure 4.

Supplementary Note 3: Optimally efficient geometry

The total efficiency of our achromatic polarisation converter is limited by the in- and out-coupling method. Standard polarisation coupling is not possible in this case since the polarisation of the output beam varies across the beam profile. A non-polarising beamsplitter can optimally achieve a 50% in- and out-coupling efficiency, leading in our set-up to an overall efficiency of 25%. In principle however, other coupling schemes could be employed which do not rely on polarisation, such as frequency-based coupling, or complex geometries. For example, if the input beam could be wavelength converted before impinging on the cone, then the output beam can be outcoupled using a dichroic mirror. In general however, wavelength conversion methods are inefficient therefore geometrical coupling methods seem to be the best candidate for optimal efficiency. One such geometry is shown in Supplementary Figure 5. The beam is split into two halves, each is back-reflected from a cone, and then recombined. The largest limitation of this set-up will be the diffraction experienced by the split beam, and the accuracy in recombining the two halves.
